# Natural turn measures predict recurrent falls in community-dwelling older adults: a longitudinal cohort study

**DOI:** 10.1038/s41598-018-22492-6

**Published:** 2018-03-12

**Authors:** Julia M. Leach, Sabato Mellone, Pierpaolo Palumbo, Stefania Bandinelli, Lorenzo Chiari

**Affiliations:** 1 Personal Health Systems Laboratory, Department of Electrical, Electronic, and Information Engineering “Guglielmo Marconi” (DEI), University of Bologna, Viale Risorgimento, 2, 40136 Bologna, Italy; 2 Azienda Sanitaria Toscana Centro, Firenze, Piero Palagi Hospital, Viale Michelangelo 41, 50125 Firenze, Italy

## Abstract

Although turning has been reported as one of the leading activities performed during a fall, and falls during turning result in 8-times more hip fractures than falls during linear gait, the quantity and quality of turns resulting in falls remain unknown since turns are rarely assessed during activities of daily living. 160 community-dwelling older adults were monitored for one week using smartphone technology. Turn measures and activity rates were quantified. Fall incidence within 12 months from continuous monitoring defined fall status, with 7/153 prospective fallers/non-fallers. Based on the analysis of 718,582 turns, prospective fallers turned less frequently, took longer to turn, and were less consistent in turn angle (p = 0.007, 0.025, and 0.038, respectively). Prospective fallers also walked slower and spent less time walking and turning and more time engaged in sedentary behavior (p = 0.043, 0.012, and 0.015, respectively). Individuals experiencing decline in the control of gait and/or turning may attempt to reduce their risk of falling by limiting their exposure and implementing cautionary movement strategies while turning. Since there was no difference in the overall active rate between prospective fallers and non-fallers, impaired gait and turning ability, specifically, may attribute to elevated fall risk within this cohort.

## Introduction

Safely altering locomotor trajectory (i.e., turning) is essential for functional performance and independence, as it is ubiquitous during activities of daily living [ADLs] and becomes more difficult with age due to increasing sensorimotor impairments^1,2^. Difficulty turning during gait significantly contributes to mobility impairment and subsequent disability, which often results in dependence, reduced quality of life, and falls, a leading cause of injury and subsequent death for older adults^1,2^. In a recent study on the circumstances of falling in the elderly, 13% of all real-life falls captured on video occurred during turning (7% during linear gait and turning, 6% during quiet stance and turning)^3^. Although turning has been reported as one of the leading activities performed at the time of falling^3^, and although falls during turning result in 8-times more hip fractures than falls during linear gait^4,5^, the quantity and quality of turns resulting in falls remain unknown since turns are rarely assessed during ADLs and in the comfort of one’s natural living environment^6–8^.

Turning is more complex and demanding than linear gait as it requires more neural resources to plan and coordinate postural transitions, more coupling between the balance and gait control systems, and more spatial coordination between limbs^9,10^. It is suggested that because of this, turning-related neural systems may be more vulnerable to impairment and decline compared to linear gait-related neural systems^9,10^. As such, turning assessments may be more sensitive to mobility disability and more predictive of future falls than gait assessments alone.

Research has revealed age-associated differences in both turn execution and strategy. Age-related changes in turning strategy may be due to the older adults’ loss of coordination and compensatory reliance on feedback mechanisms^11^. Although healthy older adults tend to utilize more time and steps to turn^12–14^, they often employ a turning strategy with less stability and greater biomechanical cost compared to healthy young adults^11,15,16^. Furthermore, turning strategy has been found to predict multiple falls in community-dwelling older adults: future recurrent fallers demonstrated a more simplified movement pattern compared to future non-fallers during 360° standing turns^17^. This suggests that high-risk older adults simplify their movements to reduce exposure and assist in balance control. Therefore, differences and/or changes in turning strategy may mark an increased risk of falling.

The majority of turning and fall studies have relied on either clinical^1,11^ or laboratory-based^13,17^ measures to assess turning performance. Current clinical measures are qualitative, subjective, and influenced by rater bias. Although laboratory-based measures are quantitative and objective, they depend on expensive laboratory-grade equipment and strict testing protocols within a controlled environment. Neither are feasible methods for assessing turning on a frequent basis and in the natural living environment. Since turning accounts for as much as 45% of steps taken within a day^18^ and poses an increased risk of falling^19^ compared to linear gait^20^, and since most falls occur during ADLs, characterizing turning during natural daily life is essential in order to develop a better understanding of true mobility disability and fall risk in the older adult population. Despite this, the associations between natural turning and falls remain relatively unexplored. All but one study that have assessed fall risk via activity monitoring have focused on measures of gait^21–23^ or postural transitions^24,25^. To date, only one fall study has quantified turns in the natural living environment across time. In their pilot study, Mancini *et al*.^8^ were the first and only to continuously monitor turns and investigate the associations between turning performance and fall history/risk. The authors observed many associations between turn quality (but not quantity) and fall history, as well as an association between the day-to-day variability in turn quality and future falls.

Although almost every activity of daily living requires some degree of turning^26^, not all turns are alike. Varying angular ranges of turning during different physical orientations and movements (e.g., quiet stance, gait, or a postural transition) likely demand varying amounts of neural control and rely on different motor planning strategies. A 180° turn during a postural transition (e.g., turn-to-sit) is a very conscious movement^14^ and requires more neural resources, coupling, and spatial coordination than a relatively subconscious, gradual 60° turn during gait. As such, it is important to specify both turn type (e.g., during quiet stance, gait, postural transition, etc.) and angular range (e.g., (50–100°] vs. (100–150°] vs. (150–200°]) when analyzing turns during ADLs.

The purpose of this study was to assess the relationships between natural turns and fall history/risk in community-dwelling older adults. Smartphones were worn at waist-level and used to measure a broad angular range of turns during ADLs for about one week. Turn quantity, quality, and variability were then assessed relative to fall incidence, both retrospectively and prospectively for 12 months.

## Methods

### Study design and participants

All participants involved in the 4^th^ wave of the InCHIANTI study (ClinicalTrials.gov, NCT01331512)^27^ were invited to partake in one week of continuous activity monitoring immediately following their clinical evaluation. One hundred and seventy one of those who volunteered were community-dwelling older adults over the age of 65 (mean 79·7 ± standard deviation [SD] 6·6 years, 50·9% female), scored greater than 24 on the Mini-Mental State Exam [MMSE], did not have pacemakers, and were able to perform ADLs without any assistance. These 171 participants were monitored for 5–9 days (mean 6·4 ± SD 1·2 days) between June 2013 and July 2014 using smartphone technology. An observation time of approximately seven days was decided as a compromise between the number of devices available, the number of participants to be assessed each month, and the minimum number of days needed to characterize physical activity and sedentary behavior in older adults^28^.

An Android smartphone, with the uFALL app^29^ pre-installed, along with an elastic case waist belt was provided to the participants by the InCHIANTI clinical staff, who also provided a practical demonstration on how to use and charge the device. Once comfortable with the smartphone technology, participants brought the device home, used it for one week, and then returned it to the clinical staff at the end of the monitoring period. The smartphone, embedded with a tri-axial accelerometer and gyroscope, was worn on the midsagittal plane of the lower back during all waking hours and was used to continuously monitor daily activity patterns. A validated turn detection algorithm^6^, based on the angular rotational rate of the pelvis about the vertical axis, was used to detect turns. Relative turn angles were obtained by numerical integration. All turns with angles between 50–200° and durations between 0·5–5 seconds were subject to analysis if they occurred during gait (i.e., turns that met the specified angle and duration thresholds but occurred during quiet stance or a postural transition were excluded). Participants were included in the analysis if they had at least five full days-worth (i.e., at least 12 hours per day) of continuous monitoring and if they executed at least ten large (150–200°] turns within the first three days.

The study protocol was approved by the ethical committee of the Italian National Institute of Research and Care of Aging and complies with the Declaration of Helsinki. All participants received a detailed description of the study purpose and procedures and gave their written informed consent.

### Turn quantity, quality, and variability measures

Turn quantity was characterized by the number of turns per (monitoring) hour (*TPH*). Turn quality was characterized by the turn angle (*ANG*), turn duration (*DUR*), mean and peak turn velocity (*MV, PV*), the logarithm of the normalized jerk score (*NJS*), and the number of steps to turn (*NSTEP*). The daily means, overall means (quantified by the mean of all turns across the monitoring period), and the day-to-day variability (quantified by the coefficient of variation [CoV] of the daily means across all monitoring days) were calculated for all seven turn measures.

The *NJS* measure represents turning smoothness, normalized by both turn *ANG* and *DUR*, and was derived from the second derivative of the turn’s angular velocity, as detailed in Caby *et al*.^30^. The *NSTEP* measure was derived using the anterior-posterior acceleration signal, as detailed in Zijlstra *et al*.^31^. We quantified turn complexity with our *NSTEP* and *NJS* measures: human motion is considered more complex if it involves more coordination (e.g., more steps) and the temporal movement is less smooth (i.e., more jerky)^32^. We quantified the size of the turn with our *ANG* measure and the length/speed of the turn with our *DUR, MV*, and *PV* measures.

### Gait speed and activity rate measures

Gait speed was quantified by the time to complete the 7-Meter Walk Test and was measured during a clinical evaluation (ClinicalTrials.gov, NCT01331512)^27^ performed a few days prior to continuous monitoring. Activity rates were derived from the continuous monitoring dataset and used to represent the percentage of time spent engaged in specific activities throughout the monitoring period: 1. Gait rate, which includes turning during gait; 2. Active rate, which includes all active (i.e., non-sedentary) behavior other than gait; and, 3. Sedentary rate, which includes all sedentary (i.e., non-active) behavior.

### Retrospective/prospective fall status

Fall incidence within 12 months from continuous monitoring defined fall status, both retrospectively and prospectively. Participants were asked to report retrospective falls that had occurred during the 12 months prior during their clinical evaluation. A monthly telephone interview was conducted to ascertain the occurrence of prospective falls during the 12 months post. Participants who did not fall or fell just once were defined as non-fallers [NFs] and participants who fell more than once (i.e., recurrently) were defined as fallers [Fs].

### Statistical analysis

The turning dataset was first divided into three subsets based on turn angle (small (50–100°], medium (100–150°], and large (150–200°]) to account for different motor planning strategies within our analysis. Univariate logistic regression was then performed to assess the relationships between the turn measures (derived for the three distinct turn angle ranges), gait speed, activity rates and fall incidence, both retrospectively and prospectively. Both unadjusted and adjusted estimates (odds ratios [OR]) and their precision (95% confidence intervals [CI]) were calculated from the z-scores of all measures. Robust linear regression was used to remove the effect of age, sex, weight, height, and MMSE on turning ability^33,34^. Statistical significance was defined at *p* < 0.05. False discovery proportion estimation by permutation was employed to account for repeated testing^35^. The 95% upper bound was calculated with the approximate method of the closed testing procedure with 10,000 random permutations.

Most all data was processed and analyzed using MATLAB R2015a (MathWorks, Natick, MA, USA), with the exception of the false discovery proportion estimation, which was calculated using the “confSAM” R Package (R Core Team, Version 3.3.2 (2016), Vienna, Austria) by Hemerik and Goeman^35^.

### Data availability

The data that support the findings of this study are available from the InCHIANTI study but restrictions apply to the availability of these data, which were used under license for the current study, and so are not publicly available. Data are however available from the authors upon reasonable request and with permission of the InCHIANTI study.

## Results

### Fall status

**Figure 1:**
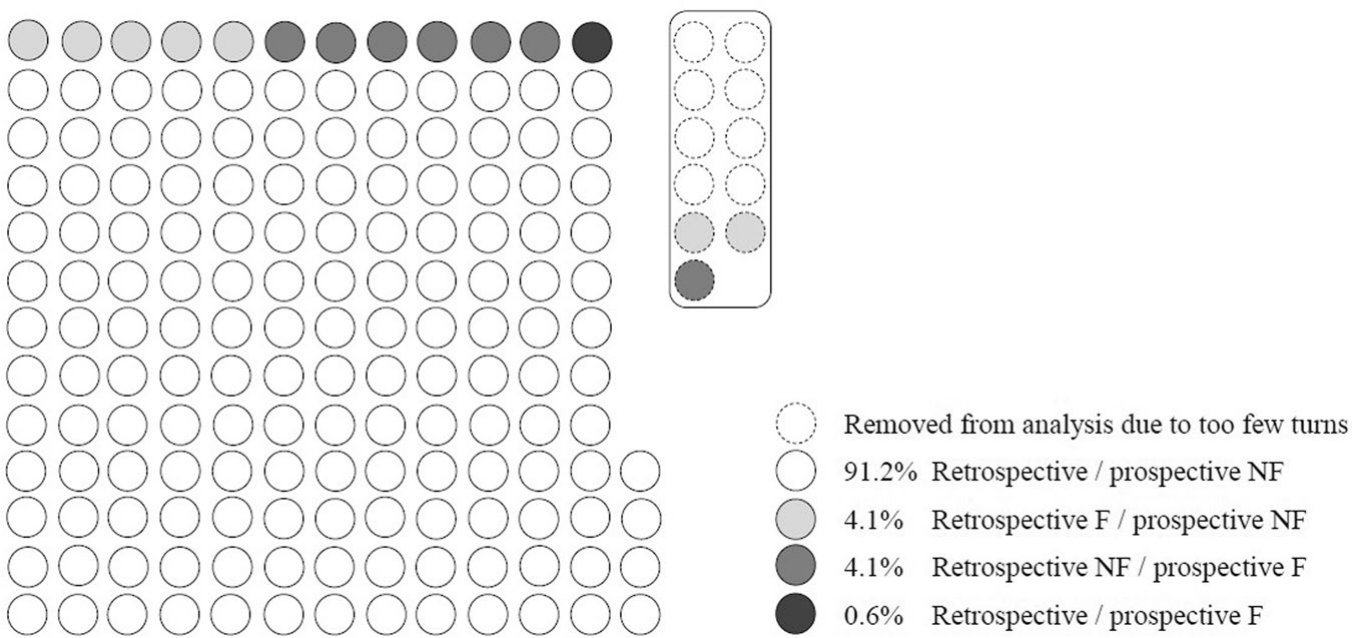
Number and percentage of participants reporting 12-month retrospective and/or prospective falls. Retrospective/prospective fall status is illustrated for all 171 participants. Eleven participants were excluded from the analysis due to too few turns. With 11 participants removed, 160 participants remained: 154 non-fallers [NFs]/6 fallers [Fs] retrospectively and 153 NFs/7 Fs prospectively.

The large majority of participants (156/171: 91·2%) had no history of recurrent falls and no future recurrent falls (retrospective/prospective NFs). Seven participants experienced recurrent falls during the 12 months prior to continuous monitoring but did not experience future recurrent falls (retrospective Fs/prospective NFs). Another seven participants had no history of recurrent falls but did experience future recurrent falls during the 12 months following continuous monitoring (retrospective NFs/prospective Fs). One participant had both a history of recurrent falls and experienced future recurrent falls (retrospective/prospective F). See Fig. 1 for an illustration of the distribution of fall status classification across all 171 participants.

Eleven participants (eight retrospective/prospective NFs, two retrospective Fs/prospective NFs, and one retrospective NF/prospective F) did not meet the inclusion criteria. One hundred and sixty participants (154 NFs/6 Fs retrospectively and 153 NFs/7 Fs prospectively) remained after these 11 participants were excluded from the analysis. Figure 1 details which participants were included/excluded from the analysis.

### Natural turning characteristics

**Figure 2:**
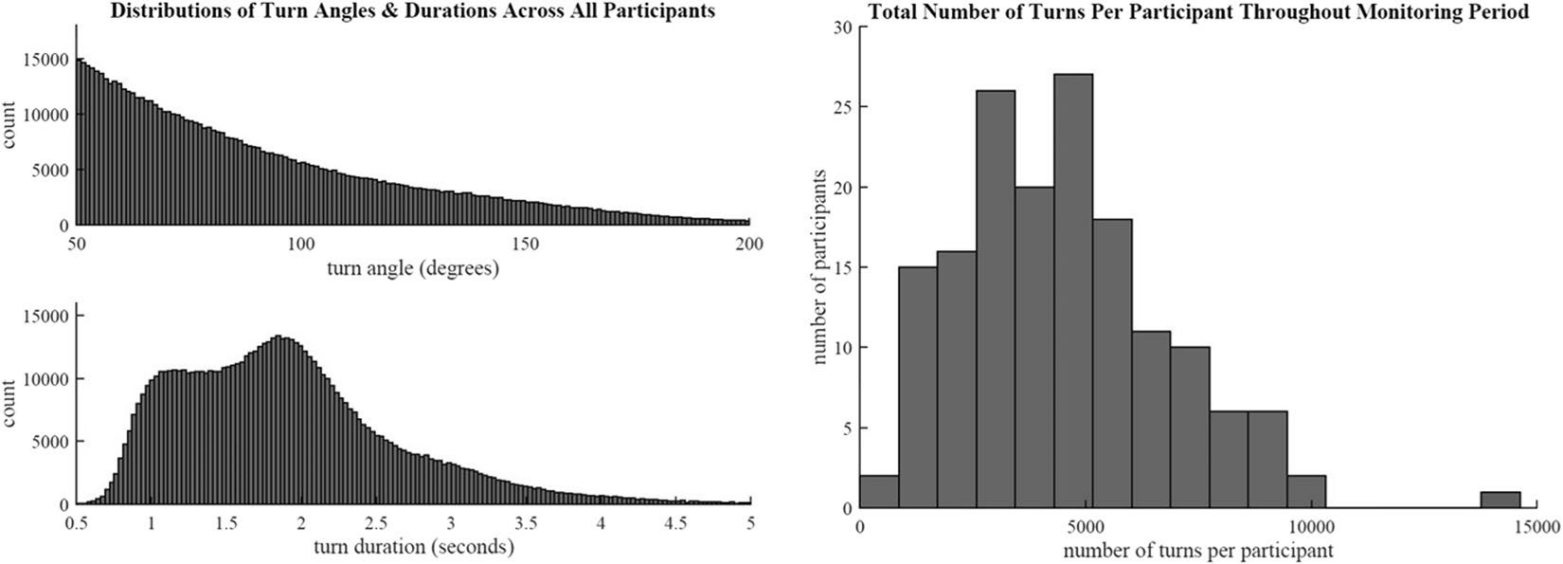
Distributions of turn angles, turn durations, and total number of turns per participant across the monitoring period. These histograms show all evaluated turns (n = 718,582) acquired from the 160 participants included in this analysis. The majority of evaluated turns were smaller (median, 81.74°) and lasted under 2 seconds (median, 1.81 s). On average, each participant performed around 5,000 turns throughout the 5–9 days of monitoring.

The 160 selected participants produced a total of 718,582 evaluated turns. The distributions of both turn angle and duration are illustrated in Fig. 2, with a mean angle of 90·91 ± SD 33·85° and mean duration of 1·90 ± SD 0·74 s. Small, medium, and large turns accounted for 67·5% (n = 484,853), 24·9% (n = 179,039), and 7·6% (n = 54,690) of all evaluated turns, respectively. The distribution of the total number of turns per participant is also illustrated in Fig. 2, with a mean of 4,491 ± SD 2,281 turns per participant.

Summary statistics for the natural turn measures are reported in Table 1, averaging across all participants regardless of fall status. Both the overall means and day-to-day variability of each turn measure are reported for each of the three turn angle subsets. For example, the overall mean of *DUR* reported for the (50–100°] turn angle subset represents the average duration of all turns with turn angles >50° and ≤100°.

**Table 1:** Summary statistics for natural turn measures: overall means and day-to-day variability.

Turn Measures	Units*	(50–100°]		(100–150°]		(150–200°]	
		Overall Means Mean ± SD	Day-to-Day Variability Mean ± SD	Overall Means Mean ± SD	Day-to-Day Variability Mean ± SD	Overall Means Mean ± SD	Day-to-Day Variability Mean ± SD
*DUR*	*s*	1.71 ± 0.25	0.03 ± 0.02	2.42 ± 0.34	0.03 ± 0.02	3.04 ± 0.40	0.05 ± 0.02
*MV*	*°/s*	45.42 ± 6.54	0.03 ± 0.04	53.81 ± 7.53	0.04 ± 0.05	58.62 ± 8.10	0.05 ± 0.02
*PV*	*°/s*	82.75 ± 10.09	0.03 ± 0.03	99.04 ± 12.11	0.04 ± 0.04	109.34 ± 13.37	0.04 ± 0.02
*NJS*	—	5.79 ± 0.37	0.02 ± 0.01	6.34 ± 0.36	0.02 ± 0.01	6.75 ± 0.36	0.02 ± 0.01
*NSTEP*	**—**	2.67 ± 0.50	0.04 ± 0.02	3.90 ± 0.70	0.04 ± 0.02	4.99 ± 0.84	0.06 ± 0.03
*TPH*	*/h*	35.90 ± 16.79	0.22 ± 0.10	13.41 ± 7.23	0.25 ± 0.11	4.26 ± 2.81	0.30 ± 0.14

Means and standard deviations [SD] are reported.

*Units are specified for the overall means; the day-to-day variability is quantified by the coefficient of variation [CoV], a unit-less value.

### Associations between natural turning and retrospective/prospective falls

Only two associations between natural turning and retrospective/prospective falls were observed before adjusting for age, sex, weight, height, and MMSE: 1. More day-to-day variability in the turn quantity (quantified by *TPH*) of larger turns was associated with retrospective Fs; and, 2. More day-to-variability in the turn quality (quantified by *ANG*) was associated with prospective Fs.

**Figure 3:**
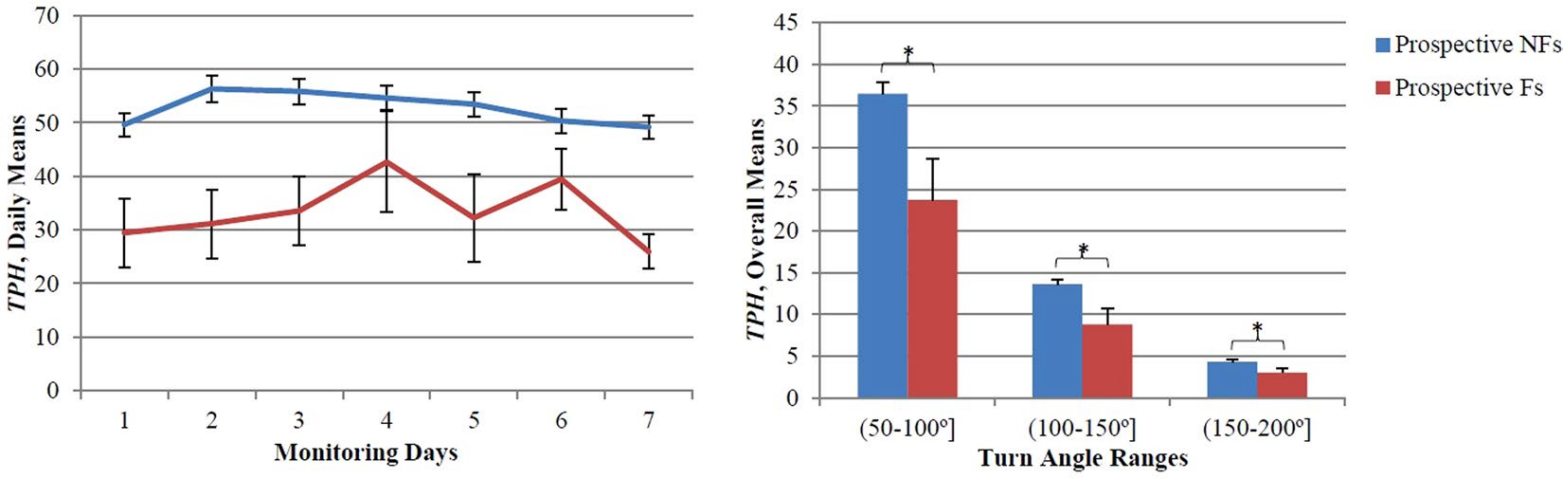
Lower turn quantity differentiates future recurrent fallers from non- and single-fallers. In the left panel, the daily group means and standard errors [SEs] of *TPH* for all evaluated turns are plotted across one week of continuous monitoring. Prospective Fs (red) consistently turned less frequently throughout the day compared to prospective NFs (blue) (from Table 2, *p* = 0.007). In the right panel, the overall group means and SEs of *TPH* are plotted for all three turn angle ranges. Prospective Fs turned less frequently than prospective NFs, regardless of turn angle (from Table 2, *p* = 0.007, 0.011, and 0.046 for the small, medium, and large turns, respectively).

**Figure 4:**
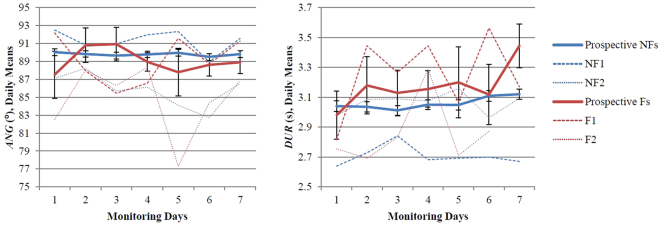
More day-to-day variability in turn angle and duration (of larger turns) differentiates future recurrent fallers from non- and single-fallers. The daily group means and SEs of *ANG* for all evaluated turns (left panel) and *DUR* for larger turns (right panel) are plotted across one week of continuous monitoring. Although there is no difference in *ANG* and *DUR* (for larger turns) between groups, prospective Fs (red) have significantly more day-to-day variability in *ANG* and *DUR* (for larger turns) compared to prospective NFs (blue) (from Table 2, *ANG, (50–200°]*: *p* = 0.038, *DUR, (150–200°]: p* = 0.034). Two participants from each group (prospective NFs: NF1, NF2; and, prospective Fs: F1, F2) are plotted to show the difference in the day-to-day variability between individual participants: the recurrent future fallers are less consistent in both the angular size of turning and the timing of large turning across days.

The adjusted results derived from the complete turning dataset, as well as from the three distinct turn angle subsets, are reported in Table 2. The natural turn measures were only associated with prospective (but not retrospective) fall status when analyzing across all turns. Fewer *TPH* (Fig. 3), longer *DUR*s, and more day-to-day variability in *ANG* (Fig. 4) were associated with prospective Fs. When differentiating based on turn angle ranges, more day-to-day variability in the turn quantity (of larger turns) was associated with retrospective Fs and lower turn quantity and quality, as well as more day-to-day variability in turn quality, was associated with prospective Fs. Participants who fell more than once during the 12 months following continuous monitoring turned less frequently (fewer *TPH*, across all three turn angle ranges, Tables 2 and 3, Fig. 3) compared to participants who did not fall or fell just once. These participants also demonstrated longer *DUR*s, lower *MV*s and *PV*s, and more *NSTEP*s while executing smaller turns (Tables 2 and 3) and were less consistent in *DUR* while executing larger turns (Fig. 4) compared to future non-/single-fallers.

**Table 2:** The associations between natural turns and retrospective/prospective falls.

Turn Measures	Turn Angle Ranges						All Turns	
	(50–100°]		(100–150°]		(150–200°]		(50–200°]	
	Overall Means OR (95% CI)	Day-to-Day Variability OR (95% CI)	Overall Means OR (95% CI)	Day-to-Day Variability OR (95% CI)	Overall Means OR (95% CI)	Day-to-Day Variability OR (95% CI)	Overall Means OR (95% CI)	Day-to-Day Variability OR (95% CI)
Retrospective Falls								
*ANG*	0.64 (0.31–1.31)	1.06 (0.48–2.31)	**0.44 (0.19–0.99)**	0.89 (0.35–2.22)	0.63 (0.31–1.28)	1.24 (0.78–1.97)	0.54 (0.26–1.13)	1.05 (0.51–2.19)
*DUR*	0.88 (0.37–2.10)	1.21 (0.73–1.98)	0.98 (0.43–2.24)	1.24 (0.75–2.05)	0.79 (0.34–1.83)	1.33 (0.70–2.50)	0.81 (0.34–1.97)	1.14 (0.66–1.96)
*MV*	1.08 (0.48–2.46)	1.11 (0.67–1.86)	0.91 (0.40–2.07)	1.03 (0.50–2.11)	1.10 (0.49–2.46)	1.31 (0.66–2.61)	0.99 (0.44–2.25)	1.12 (0.69–1.84)
*PV*	0.94 (0.42–2.14)	1.14 (0.69–1.87)	0.87 (0.38–1.99)	1.11 (0.66–1.85)	0.96 (0.42–2.19)	1.48 (0.74–2.93)	0.87 (0.38–1.99)	1.16 (0.74–1.83)
*NJS*	0.67 (0.29–1.59)	1.08 (0.55–2.12)	0.88 (0.38–2.03)	0.60 (0.13–2.71)	0.74 (0.32–1.73)	1.08 (0.49–2.35)	0.66 (0.028–1.56)	0.94 (0.36–2.44)
*NSTEP*	0.92 (0.39–2.16)	0.98 (0.42–2.30)	1.04 (0.46–2.33)	0.91 (0.36–2.29)	0.93 (0.41–2.12)	1.63 (0.87–3.04)	0.89 (0.38–2.10)	0.90 (0.33–2.46)
*TPH*	1.00 (0.44–2.27)	1.18 (0.54–2.58)	0.75 (0.30–1.88)	1.49 (0.75–2.97)	0.60 (0.22–1.65)	**1.92 (1.07–3.44)**	0.89 (0.38–2.10)	1.27 (0.58–2.79)
Prospective Falls								
ANG	0.80 (0.39–1.64)	**1.88 (1.10–3.18)**	1.52 (0.81–2.81)	1.12 (0.57–2.20)	**0.40 (0.21–0.76)**	**1.79 (1.01–3.18)**	0.92 (0.44–1.94)	**1.87 (1.04–3.37)**
*DUR*	**2.04 (1.16–3.61)**	1.13 (0.66–1.95)	1.64 (0.85–3.14)	1.33 (0.87–2.05)	1.52 (0.73–3.19)	**1.74 (1.04–2.89)**	**2.03 (1.09–3.76)**	1.05 (0.54–2.02)
*MV*	**0.44 (0.20–0.94)**	0.98 (0.43–2.25)	0.60 (0.28–1.31)	1.17 (0.79–1.73)	0.62 (0.28–1.38)	1.41 (0.76–2.61)	0.49 (0.22–1.08)	1.17 (0.78–1.75)
*PV*	**0.45 (0.20–0.99)**	0.82 (0.20–3.40)	0.53 (0.23–1.18)	1.10 (0.66–1.82)	0.54 (0.23–1.24)	1.67 (0.90–3.10)	0.48 (0.21–1.07)	0.94 (0.36–2.45)
*NJS*	2.09 (0.99–4.41)	1.18 (0.71–1.96)	1.40 (0.68–2.87)	1.37 (0.93–2.03)	1.16 (0.54–2.45)	1.49 (0.81–2.74)	1.86 (0.88–3.97)	1.19 (0.75–1.90)
*NSTEP*	**1.80 (1.01–3.22)**	1.23 (0.76–2.00)	1.50 (0.75–2.99)	1.45 (0.91–2.31)	1.36 (0.64–2.87)	1.62 (0.90–2.92)	1.76 (0.92–3.37)	1.24 (0.77–1.99)
*TPH*	**0.17 (0.05–0.62)**	1.88 (0.96–3.68)	**0.19 (0.05–0.68)**	1.85 (0.99–3.44)	**0.31 (0.10–0.98)**	1.23 (0.69–2.19)	**0.17 (0.05–0.62)**	2.05 (1.00–4.22)

Odds ratios [OR] (95% confidence intervals [CI]) from the adjusted univariate logistic regressions are reported. Bolded values represent statistical significance at a *p* < 0.05. *P*-values have not been adjusted for multiple comparisons.

**Table 3:** Difference in overall means between prospective fallers (Fs) and non-fallers (NFs).

Turn Measures	Units*	Overall Means								
		(50–100°]			(100–150°]			(150–200°]		
		NFs Mean ± SD	Fs Mean ± SD	p	NFs Mean ± SD	Fs Mean ± SD	p	NFs Mean ± SD	Fs Mean ± SD	p
*DUR*	*s*	**1.71 **±** 0.24**	**1.90 **±** 0.47**	**0.014**	2.42 ± 0.34	2.56 ± 0.40	0.138	3.04 ± 0.40	3.14 ± 0.39	0.264
*MV*	*°/s*	**45.58 **±** 6.39**	**41.97 **±** 9.27**	**0.034**	53.91 ± 7.52	51.52 ± 8.10	0.200	58.72 ± 8.10	56.43 ± 8.38	0.240
*PV*	*°/s*	**82.96 **±** 9.83**	**78.17 **±** 14.92**	**0.047**	99.23 ± 11.88	94.77 ± 17.01	0.120	109.54 ± 13.14	104.93 ± 18.47	0.147
*NJS*	**—**	5.78 ± 0.37	6.01 ± 0.46	0.053	6.34 ± 0.37	6.43 ± 0.32	0.364	6.75 ± 0.37	6.77 ± 0.32	0.707
*NSTEP*	**—**	**2.66 **±** 0.46**	**3.00 **±** 1.05**	**0.047**	3.88 ± 0.68	4.14 ± 1.05	0.255	4.99 ± 0.83	5.19 ± 1.11	0.423
*TPH*	**/** *h*	**36.45 **±** 16.77**	**23.73 **±** 12.99**	**0.007**	**13.62 **±** 7.25**	**8.82 **±** 5.18**	**0.011**	**4.32 **±** 2.84**	**3.00 **±** 1.44**	**0.046**

Group means and standard deviations [SD] are reported. Bolded values represent statistical significance at a *p* < 0.05 (derived from the adjusted univariate logistic regressions). *P*-values have not been adjusted for multiple comparisons.

Results from our calculations on the false discovery proportion estimation by permutation are as follows. For retrospective falls, there were two rejections at *p* < 0.05 (Table 2) and the median estimate and 95% upper bound for the number of false discoveries was one and two, respectively. For prospective falls, there were 14 rejections at *p* < 0.05 (Table 2) and the median estimate and 95% upper bound for the number of false discoveries was one and ten, respectively.

### Associations between gait and activity measures and retrospective/prospective falls

Both the unadjusted and adjusted results are reported in Table 4. Unadjusted gait speed was associated with retrospective Fs (and no associations were observed prospectively). Adjusted gait speed, gait rate, and sedentary rate were associated with prospective Fs. Future recurrent fallers took longer to complete the 7-Meter Walk and spent less time walking and more time engaged in sedentary behavior compared to future non-/single-fallers. The time spent engaged in all other active behavior excluding gait, however, was not associated with prospective fall status. There were no associations observed retrospectively after adjusting for age, sex, weight, height, and MMSE.

**Table 4:** Associations between other activity measures and retrospective/prospective falls.

Other Activity Measures	Unadjusted		Adjusted	
	OR (95% CI)	p	OR (95% CI)	p
Retrospective Falls				
*Gait Speed*	**0.38 (0.16–0.89)**	**0.026**	0.43 (0.16–1.12)	0.083
*Gait Rate*	0.91 (0.40–2.10)	0.832	1.05 (0.47–2.36)	0.908
*Active Rate*	2.02 (0.91–4.47)	0.085	1.93 (0.90–4.13)	0.091
*Sedentary Rate*	0.72 (0.32–1.64)	0.436	0.67 (0.30–1.48)	0.322
Prospective Falls				
*Gait Speed*	0.59 (0.26–1.35)	0.214	**0.36 (0.13–0.97)**	**0.043**
*Gait Rate*	**0.34 (0.13–0.92)**	**0.034**	**0.23 (0.08–0.73)**	**0.012**
*Active Rate*	0.53 (0.22–1.27)	0.157	0.52 (0.21–1.27)	0.148
*Sedentary Rate*	**2.61 (1.09–6.28)**	**0.032**	**3.67 (1.29–10.48)**	**0.015**

Odds ratios [OR] (95% confidence intervals [CI]) from both the unadjusted and adjusted univariate logistic regressions are reported. Bolded values represent statistical significance at a *p* < 0.05. *P*-values have not been adjusted for multiple comparisons.

## Discussion

In this study, we illustrate the importance of continuous monitoring as a means to characterize turning and its association with fall risk in older adults. Turn quantity and quality, as well as the day-to-day variability in turn quality, were found to be sensitive to prospective fall status. Older adults who endured more than one fall within the 12 months following continuous monitoring turned less frequently, took longer to turn, and varied more in angular turn size across days compared to older adults who did not fall or fell just once. Additionally, lower turn quality (quantified by longer durations, lower velocities, and more steps) during smaller turns and more day-to-day variability in the duration of larger turns were associated with future recurrent falls. These findings suggest that continuous monitoring methods may be essential when working to predict future falls since information on turn quantity, quality, and variability across time is only obtainable by use of continuous monitoring via a body-worn sensor.

Both Thigpen *et al*.^11^ and Wright *et al*.^17^ have substantiated evidence suggesting that older adults at a higher risk of falling employ a simplified movement pattern during turning to assist in balance control and prevent a fall. According to Spirduso *et al*.^36^, older adults often adopt a strategy consisting of simpler, smaller, and/or slower movements to compensate for coordination loss. Although turning strategy could not be fully characterized via our continuous monitoring methods since body segment kinematic analysis requires the use of more than one sensor, the turn quality measures obtained in this study can be used to characterize certain features of turning strategy, as detailed in our Methods. Unlike findings from prior studies^1,8^, turn complexity, size, and length/speed were not associated with a history of recurrent falls in this study (Table 2). We did, however, observe associations between lower turn quality and future recurrent falls.

Future recurrent fallers took longer to turn, turned slower, and took more steps to turn while executing small turns compared to future non-/single-fallers (Tables 2 and 3). This may suggest that longer turn durations, slower turn velocities, and more steps taken during a turn are indicative of a cautionary movement strategy. Future recurrent fallers may turn more carefully during the most common angular turn range (i.e., small turns: (50–100°]) since older adults at an elevated risk of falling have been shown to increase caution during ADLs to protect against falls^37^. There were no differences in turn quality between future recurrent fallers and non-/single-fallers while executing the medium and large turns (Tables 2 and 3). An older adult with declining stability will likely pay close attention during larger turns since the risk of falling during a turn increases as turn size increases^20^. The successful execution of larger turns requires a significant amount of neural resources, coupling, and spatial coordination^9,10^. Conversely, smaller turns are thought to be less demanding, both physically and cognitively. Since smaller turns made up the majority of the turns performed throughout the day (67·5%), and since any frequent task likely relies more on automation and less on intention, it is logical to infer that smaller turns utilize less attentional control than larger turns. Underlying instabilities are more often exposed when less attention is exerted. Therefore, turn features extracted from situations with less attentional control (i.e., during the execution of smaller turns) may be most sensitive to declining turning ability and increased fall risk during early-stage functional decline.

Lower turn quantity was also sensitive to fall risk in this study. Fewer turns per hour consistently emerged as a predictor of future recurrent falls, regardless of the angular range (Tables 2 and 3, Fig. 3). Furthermore, both gait speed and gait rate were inversely related to the incidence of future falls in this study (Table 4), pairing well with previous findings^21,23,24^. Future recurrent fallers walked slower and spent less time walking and turning (and more time engaged in sedentary behavior) compared to future non-/single-fallers. However there was no difference in the overall active rate between future recurrent fallers and non-/single-fallers (Table 4), suggesting that impaired gait and turning ability, specifically, may attribute to the older adults’ increased risk of falling within this cohort. Individuals experiencing decline in the control of gait and/or turning may attempt to reduce their risk of falling by limiting their exposure (i.e., restricting the frequency in which they walk and turn throughout the day).

We also found more day-to-day variability in turn quality to be associated with prospective fall status. Future recurrent fallers were more variable in the angular size of turning across days compared to future non-/single-fallers (Table 2, Fig. 4). Additionally, more day-to-day variability in the duration of large turns was associated with future recurrent fallers (Table 2, Fig. 4). These findings couple well with Mancini *et al*.^8^ (since they observed associations between more day-to-day variability in the number of steps per turn and future recurrent falls) and suggest that individuals with declining turning ability may be less consistent in their movement patterns and may have less consistent turning strategies in situations that demand more neural control and motor planning. In a cross-sectional study on the effect of age on turning strategy, Meinhart-Shibata *et al*.^13^ found older women to be more variable in their turn execution compared to younger women. Furthermore, models of pathologic aging assign variable behaviors to declining systems; instead of abrupt failure, motor control systems often demonstrate an initial period of increased variability during the depreciation of physiologic reserve^38^. Since increased variability in gait quality has been shown to associate with, precede, and even predict functional decline^39,40^, it is plausible that increased variability in turn quality may also be indicative of underlying pathologies. Assessing the variability patterns of motor control (e.g., gait, turning, etc.) across time remains a relatively new topic of interest with great potential in the translational research realm.

Due to this study’s exploratory nature and its relatively large number of analyzed measures, the significance of our results may be up for debate. Although we did not adjust our *p*-values to account for repeated testing, which is acceptable practice in exploratory research^22,41^, we did estimate false discovery proportions by permutation to evaluate the weight of our findings^35,42^. As previously discussed, the bulk of our findings were observed prospectively. Table 2 shows 14 significant associations between turning features and prospective falls. The number of rejections at *p* < 0.05 (14) was greater than the 95% upper bound for the number of false discoveries (10) and the median estimate of false discoveries was low (1), which reinforces our results.

The low prevalence of falls within our sample population limits our capabilities with regards to fall prediction. Only eight out of 171 participants experienced recurrent falls, both retrospectively and prospectively. Even if we were to soften our definition of a “faller” and include participants who experienced a single fall within the 12 months before or after continuous monitoring, we would still only have 28 (16·4%) retrospective and 29 (17·0%) prospective fallers. The prevalence of falls within our cohort is approximately half that of the global average (28–35% of community-dwelling older adults over the age of 64 fall at least once annually)^43^, suggesting that our cohort is relatively healthy and physically fit compared to “normal.” Our participants were recruited from the InCHIANTI study, an ongoing longitudinal population-based study aimed at identifying measures that clinicians can use to understand the causes of gait impairments in older adults^27^. All 171 participants have been active InCHIANTI participants for years and volunteered their time because they were comfortable with our testing protocol. Perhaps, due to the demands of our methodology and the nature of this study itself, we pre-selected/recruited a healthier, more active and physically capable sample population. To test the significance of our results, our methods should be applied to a cohort that is more representative of the global average. Consciously recruiting a heterogeneous sample consisting of a more diverse spread of physical capabilities and impairments would allow us to test the validity of our findings. Additionally, obtaining a cohort with a higher fall incidence would strengthen our statistical capabilities and enable a more in-depth analysis on the relationship between turns and fall risk. As is, we have a limited at-risk population which limits the statistical power in our analysis of fall risk. Conversely though, this cohort is ideal when working to identify early markers of mobility disability. Since turning features were predictive of future recurrent falls in this low-risk population, we anticipate our turning assessment to be sensitive to early-stage manifestations of mobility impairment.

Continuous monitoring not only yields objective measurements of turn quality, but it also enables the quantification of both turn frequency and variability across time, behavior that has remained relatively unexplored until now. Characterizing natural turning during daily activities via continuous monitoring methods has great potential and will address a critical barrier to clinical practice: our end-goal is to identify a sensitive, digital marker of mobility disability that may enable early detection of increased fall risk and, in turn, yield opportunities for intervention, treatment, compensation, coping, sustained independence, and prevention of irreversible damage.
